# Analysis of the potential molecular biology of triptolide in the treatment of diabetic nephropathy: A narrative review

**DOI:** 10.1097/MD.0000000000031941

**Published:** 2022-12-02

**Authors:** Ying Gao, Zhaoan Guo, Yingying Liu

**Affiliations:** a The First School of Clinical Medicine, Shandong University of Traditional Chinese Medicine, Lixia District, Jinan City, Shandong Province, China; b Department of Nephrology, Shandong University of Traditional Chinese Medicine Affiliated Hospital, Jinan, China; c The School of Clinical Medicine, Shandong University of Traditional Chinese Medicine, Lixia District, Jinan City, Shandong Province, China.

**Keywords:** diabetic nephropathy, mechanism of action, network pharmacology, triptolide

## Abstract

To explore the potential mechanism of triptolide in diabetic nephropathy (DN) treatment using network pharmacology.

**Methods:**

The main targets of triptolide were screened using the TCMSP, DrugBank, and NCBI databases, and gene targets of DN were searched using the DrugBank, DisGeNET, TTD, and OMIM databases. All of the above targets were normalized using the UniProt database to obtain the co-acting genes. The co-acting genes were uploaded to the STRING platform to build a protein-protein interaction network and screen the core acting targets. Gene ontology and Kyoto encyclopedia of genes and genomes analyses of the core targets were performed using Metascape. Molecular docking validation of triptolide with the co-acting genes was performed using the Swiss Dock platform.

**Results:**

We identified 76 potential target points for triptolide, 693 target points for DN-related diseases, and 24 co-acting genes. The main pathways and biological processes involved are lipids and atherosclerosis, IL-18 signaling pathway, TWEAK signaling pathway, response to oxidative stress, hematopoietic function, and negative regulation of cell differentiation. Both triptolide and the active site of the core target genes can form more than 2 hydrogen bonds, and the bond energy is less than -5kJ/mol. Bioinformatics analysis showed that triptolide had a regulatory effect on most of the core target genes that are aberrantly expressed in DKD.

**Conclusion:**

Triptolide may regulate the body’s response to cytokines, hormones, oxidative stress, and apoptosis signaling pathways in DN treatment by down-regulating Casp3, Casp8, PTEN, GSA3B and up-regulating ESR1, and so forth.

## 1. Introduction

Diabetic nephropathy (DN) is a major microvascular complication of diabetes mellitus (DM) and the leading cause of end-stage renal disease worldwide.^[1]^ DN is characterized by varying degrees of proteinuria and a rapid decline in renal function in the later stages, and is currently treated mainly with symptomatic treatments such as glycemic control, lipid regulation, and edema reduction.

Tripterygium wilfordii hook f., a traditional Chinese herbal medicine, can dispel wind and dampness, promote blood circulation, relieve pain, and activate blood circulation to reduce swelling. Triptolide (TP) is an epoxidized diterpene lactone compound isolated from Tripterygium wilfordii, also known as triptolide and triptolide alcohol, and is one of the main active ingredients of Tripterygium wilfordii and its toxic component. Recent studies have found that TP has anti-inflammatory, anti-tumor, and immunosuppressive effects and is widely used in tumor, rheumatological, cardiovascular, and renal diseases.^[2]^ Clinical experience has shown that TP could reduce proteinuria in DN, stabilize renal function, and have better efficacy than renin angiotensin blockers. However, the pathogenesis of DN is complex, and there are many targets of TP action; therefore, the specific mechanism and drug mechanism of TP in treating DN are not explicit.

Network pharmacology is a new discipline that selects a specific signal and designs multitarget drug molecules. Barabási first proposed the concept of network biology in 2004,^[3]^ and Hopkins formally proposed the definition of network pharmacology in 2007.^[4]^ By 2020, the total number of relevant articles published on the China Knowledge Network and PubMed reached 1944 in 1 year, which has become a popular tool for research on the basis and mechanism of traditional Chinese medicine pharmacology.^[5]^ This study aimed to analyze the targets and mechanisms of the action of TP in treating DN by network pharmacology and conduct molecular docking validation to provide a theoretical basis and experimental direction for subsequent cell and molecular experiments and promote its clinical application.

## 2. Materials and methods

### 2.1. Major databases and softwares

TCMSP, NCBI, DrugBank, DisGeNET, TTD, OMIM, Uniprot, Venny 2.1, Cytoscape 3.9, String platform, Metascape, Auto Dock Vina, GEO database, and so forth.

### 2.2. Drug and disease targets acquisition

The targets of TP were searched in the TCMSP, DrugBank, and NCBI databases with “Triptolide” as the search term, and the species was limited to “Homo sapiens.” The targets of DN were searched in the DrugBank, DisGeNET, TTD, and OMIM databases with “diabetic nephropathy” as the search term. All the above targets were gene normalized using the UniProt database, and the drug targets and disease targets were integrated and de-weighted to obtain the TP and DN target sets.

### 2.3. Construction of protein–protein interaction (PPI) networks for the targets of action of triptolide

A PPI network was built using the string platform. The co-acting targets of TP and DN were collected using Venny 2.1 software. These were imported into the String platform to work a PPI network, set the protein species to “Homo sapiens,” hide the isolated proteins in the network, and the other parameters were kept at the default settings. The PPI network graph data were exported in TSV format. The data were imported into Cytoscape, and a network analyzer was used to analyze and calculate the network topology characteristic value of the intersection PPI and filter the data to obtain the core acting targets. Simultaneously, the protein functional sub-modules were determined by further analysis of the PPI network using the MCODE plug-in.

### 2.4. Pathway enrichment analysis

The Metascape platform integrates multiple authoritative databases and supports annotation, enrichment analysis, and construction of PPI networks for batch genes or proteins, with more powerful functions, timely updates, and reliable data. The core acting targets were analyzed using the Metascape database for gene ontology (GO) and the Kyoto Encyclopedia of Genes and Genomes (KEGG). The results were saved and passed through R software for visualization.

### 2.5. Molecular docking of TP with core acting targets

Molecular docking is an effective method to verify the relationship between molecules and targets. Molecular docking was performed using TP as the ligand and the core targets of TP for DN treatment as the receptor. The 3-dimensional structure of TP was downloaded from PubChem CID, and the core acting target structures were downloaded from the PDB database and routinely processed using Chem-Bio Drew. Then, Pymol software was used to remove redundant protein structures, remove other irrelevant ligands such as water molecules, and import Auto Dock Vina software to select the semi-flexible docking mode with docking parameters as default. A binding energy of less than 0 kJ/mol indicated that the ligand molecule could spontaneously bind to the receptor protein. And the binding energy less than –5.0 kJ/mol indicates good binding, and the smaller the binding energy, the better the docking.

### 2.6. Expression of co-acting targets in disease or triptolide intervention

Most of the therapeutic effects of drugs on diseases are achieved by directly or indirectly regulating the genes that are aberrantly expressed in the disease. Diabetic Kidney Disease and Triptolide were used as the key words to search in the GEO database, and the species was limited to “Homo sapiens.” Appropriate datasets were selected for bioinformatics analysis to clarify the abnormal expression of each co-acting target in DKD, as well as to clarify the effect of TP intervention on gene expression of co-acting targets. If the expression changes of the same gene are opposite in these 2 cases, there is a high degree of certainty that TP will work through that gene; the opposite is less certain.

## 3. Results

### 3.1. The case for drugs and disease targets

**Figure 1.**
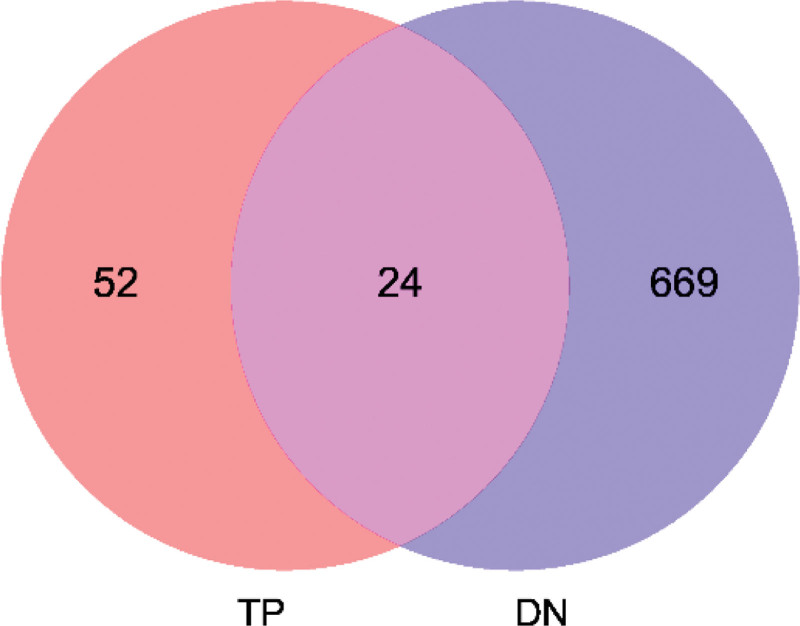
The intersection of drug and disease targets. *Note*: The red part indicates the drug targets and the blue part indicates the disease targets.

A total of 76 drug target genes, 693 disease target genes, and 24 target gene intersections were identified (Fig. 1 and Table 1).

**Table 1:** Compound-disease common targets.

No	Uniprot ID	Protien names	Gene names
1	P01024	Complement C3	C3
2	P42574	Caspase-3	CASP3
3	Q14790	Caspase-8	CASP8
4	P24864	G1/S-specific cyclin-E1	CCNE1
5	P38936	Cyclin-dependent kinase inhibitor 1	CDKN1A
6	P61073	C-X-C chemokine receptor type 4	CXCR4
7	P03372	Estrogen receptor	ESR1
8	P49841	Glycogen synthase kinase-3 beta	GSK3B
9	Q16665	Hypoxia-inducible factor 1-alpha	HIF1A
10	P11021	Endoplasmic reticulum chaperone BiP	HSPA5
11	P05231	Interleukin-6	IL6
12	O60674	Tyrosine-protein kinase JAK2	JAK2
13	P05412	Transcription factor AP-1	JUN
14	Q16236	Nuclear factor erythroid 2-related factor 2	NFE2L2
15	P00749	Urokinase-type plasminogen activator	PLAU
16	P60484	Phosphatidylinositol 3,4,5-trisphosphate 3-phosphatase and dual-specificity protein phosphatase PTEN	PTEN
17	P35354	Prostaglandin G/H synthase 2	PTGS2
18	Q04206	Transcription factor p65	RELA
19	P42224	Signal transducer and activator of transcription 1-alpha/beta	STAT1
20	P40763	Signal transducer and activator of transcription 3	STAT3
21	P01137	Transforming growth factor beta-1 proprotein	TGFB1
22	P01375	Tumor necrosis factor	TNF
23	P04637	Cellular tumor antigen p53	TP53
24	P15692	Vascular endothelial growth factor A	VEGFA

ESR = estrogen receptor, IL6 = interleukin 6, JAK = Janus kinase, STAT3 = signal transducer and activator of transcription 3, TNF = tumor necrosis factor, TP = triptolide, VEGFA = vascular endothelial growth factor A.

### 3.2. PPI network and core target genes

**Figure 2.**
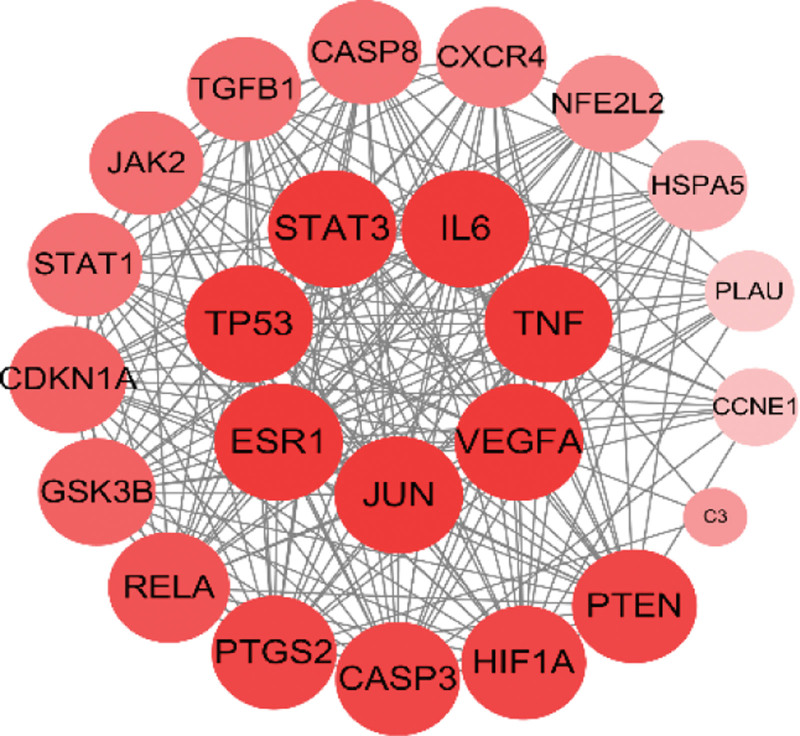
PPI network diagram of the co-acting targets. PPI = protein–protein interaction.

The co-acting targets were uploaded to the STRING platform to obtain the PPI network map. The results showed that there were 24 nodes in the network, with 220 interactions, and the average degree value was 18.3. Seven genes, including vascular endothelial growth factor A (VEGFA), tumor necrosis factor (TNF), Interleukin 6 (IL6), signal transducer and activator of transcription 3 (STAT3), cellular tumor antigen p53 (TP53), estrogen receptor (ESR1), and Transcription factor AP-1 (JUN), were associated with the highest degree value of 22; the C3 gene had the lowest degree value of 4. By combining the average degree value and median degree value, genes with degree values greater than 15 were selected as the core acting targets of TP for DN treatment. The intersection network diagram is shown in Figure 2, where the color from dark to light and the area from large to small represent the degree values from large to small. The PPI network was further analyzed using MCODE of Cytoscape with default screening parameters. Only one tightly connected protein submodule was obtained, containing 21 nodes and 195 interactions, except for C3, PLAU, and CCNE1, with a score of 19.5.

### 3.3. Visualization of enrichment analysis of pathway of TP for DN

Gene enrichment analysis was performed using the Metascape platform, including KEGG pathway analysis, biological process, cellular component, and molecular function GO analysis.

**Figure 3.**
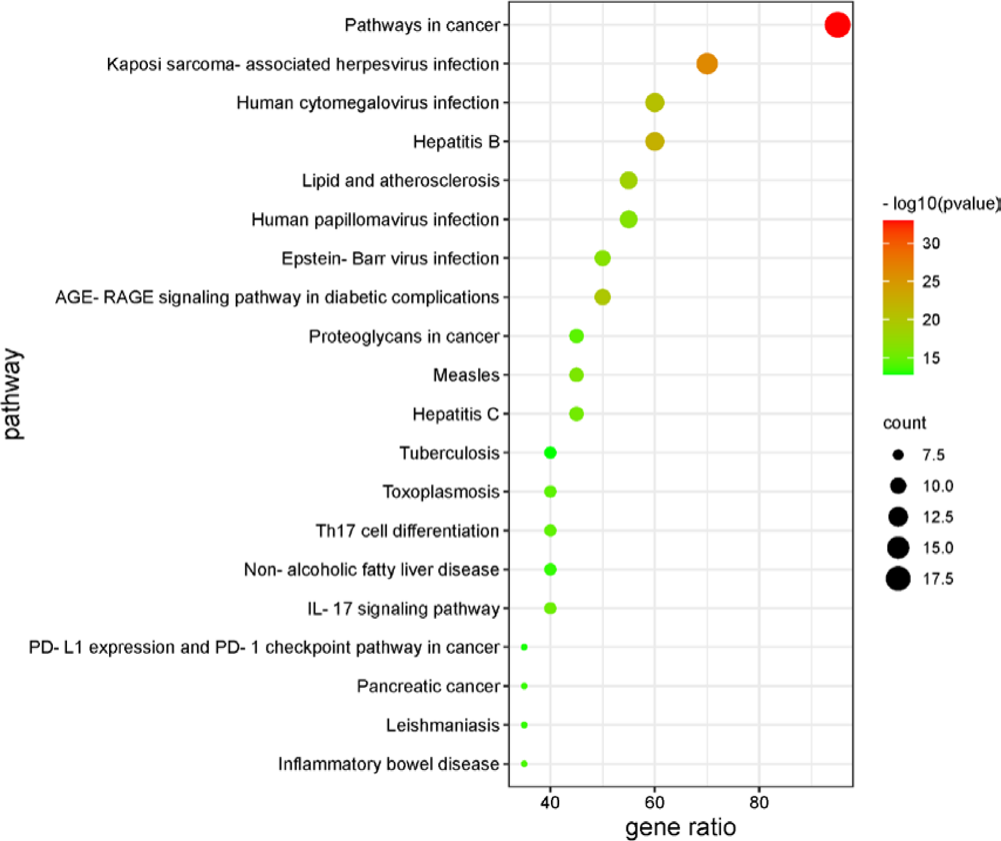
KEGG pathway analysis of the core acting targets. KEGG = Kyoto encyclopedia of genes and genomes.

After KEGG pathway analysis, the top 20 genes of Homo sapiens were sorted according to the number of enriched genes. The bubbles were plotted in *R*. The bubbles from red to green represent the -Log10 (P) values from large to small; the bubble area represents the number of genes in the pathway, and the horizontal axis represents the ratio of genes in the pathway to all genes. The results are shown in Figure 3.

**Figure 4.**
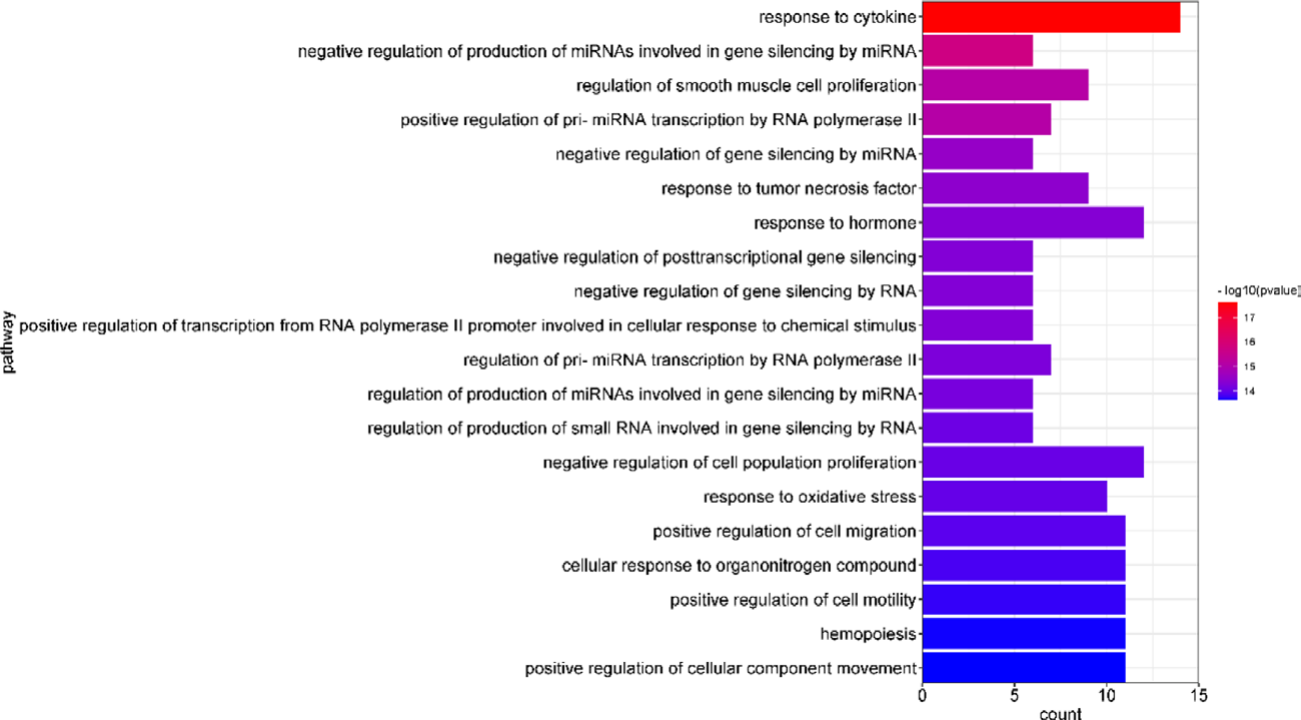
Enrichment gene ontology–biological process (GO–BP) terms for analysis of the core acting targets. BP = biological process, GO = gene ontology.

**Figure 5.**
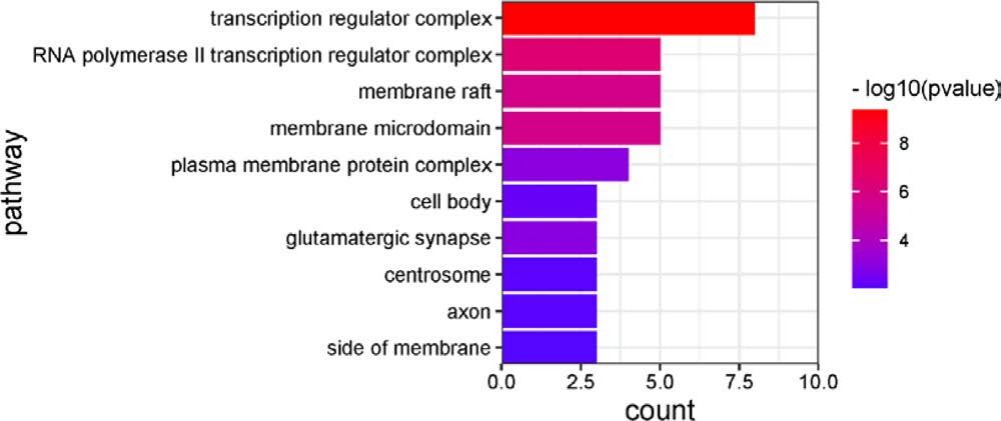
Enrichment gene ontology–cellular components (GO–CC) terms for analysis of the core acting targets. CC = cellular component, GO = gene ontology.

**Figure 6.**
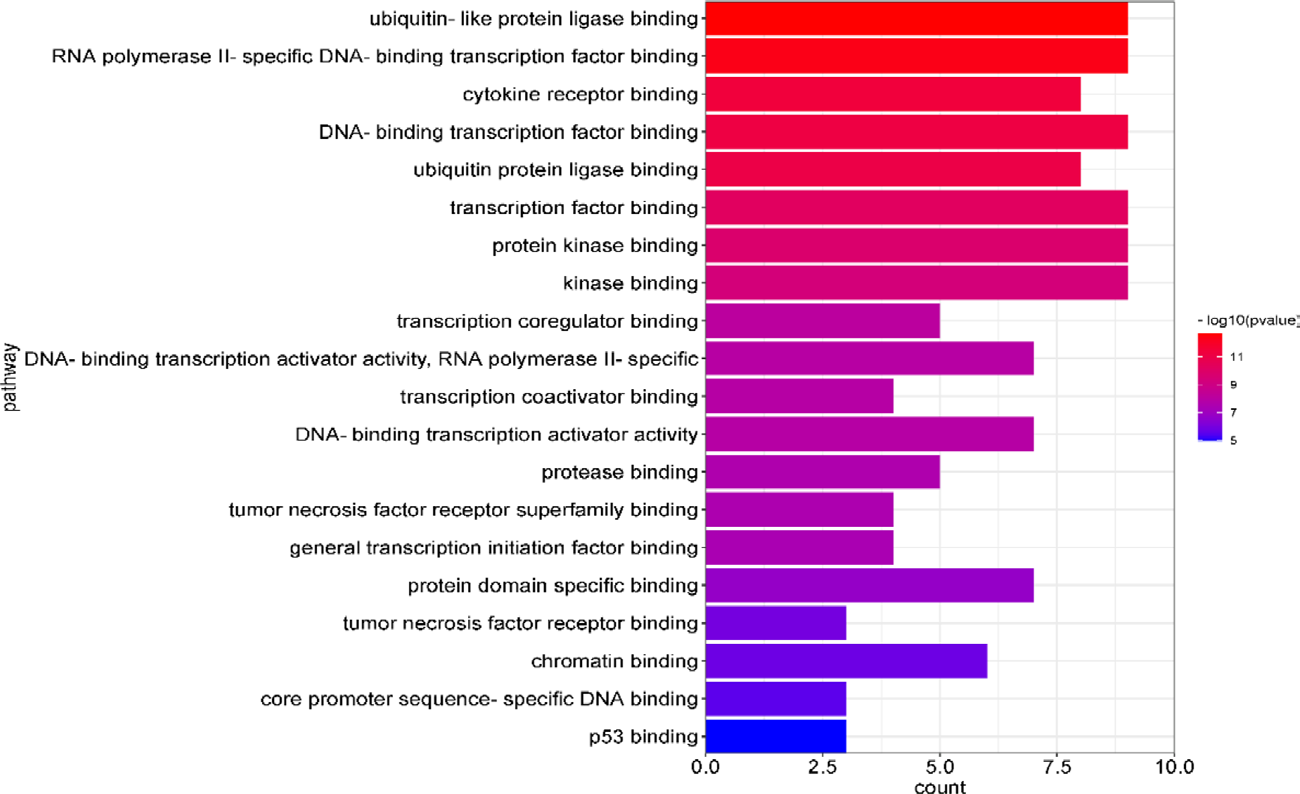
Enrichment gene ontology–molecular function (GO–MF) terms for analysis of the core acting targets. GO = gene ontology, MF = molecular function.

After GO analysis, the top 20 results with the -Log10(P) value were selected for visualization, and biological process, cellular component, and molecular function were plotted in bar graphs with the bar color from dark to light representing the -Log10(P) values from large to small and the bar length representing the gene count of that pathway. The results are shown in Figure 4–6.

### 3.4. Molecular docking of TP with core acting targets

The active sites of the core acting targets with a higher degree were able to form more than 2 hydrogen bonds, and the binding energies were all less than -5 kJ/mol, indicating good binding activity. Among them, the binding energy of tretinoin with signal transducer and activator of transcription1 was the lowest, at -7.83 kJ/mol, with the most active binding ability. Details are presented in Table 2.

**Table 2:** Molecular docking results of TP with core acting targets.

Target	Binding energy (kJ/mol)	Target	Binding energy (kJ/mol)
STAT1	–7.83	TP53	–7.42
CASP8	–7.78	PTGS2	–7.38
CXCR4	–7.77	RELA	–7.20
PTEN	–7.60	CASP3	–7.20
ESR1	–7.58	IL6	–7.16
GSK3B	–7.58	CDKN1A	–7.13
TNF	–7.50	HIF1A	–6.86
JAK2	–7.49	STAT3	–6.82
TGFB1	–7.48	VEGFA	–6.54
NFE2L2	–7.45	JUN	–6.54

ESR = estrogen receptor, JAK = Janus kinase, STAT3 = signal transducer and activator of transcription 3, TNF = tumor necrosis factor, TP = triptolide, VEGFA = vascular endothelial growth factor A.

### 3.5. Expression of co-acting targets in disease or triptolide intervention

**Figure 7.**
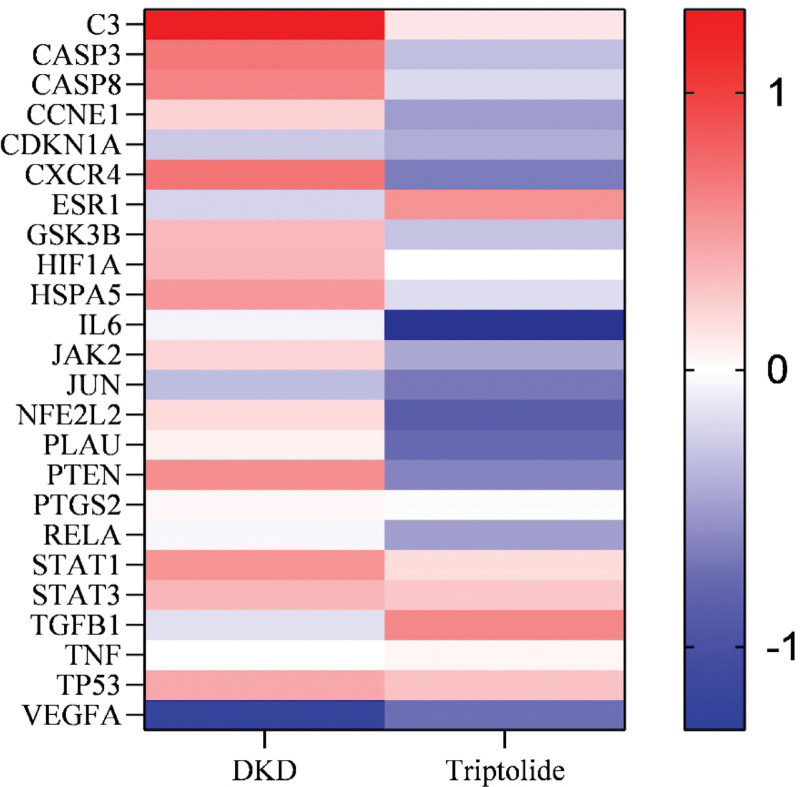
Red indicates gene up-regulation, blue indicates gene down-regulation, and the color from dark to light represents the absolute value of Log_2_FC from large to small, i.e., the expression difference is from large to small.

The GSE30122 dataset was selected for bioinformatics analysis to clarify the aberrant expression of co-acting targets in DKD, and the GSE93206 dataset was selected for bioinformatics analysis to determine the effect of TP intervention on the expression of co-acting targets by condition qualification. Red indicates gene up-regulation, blue indicates gene down-regulation, and the color from dark to light represents the absolute value of Log_2_FC from large to small, that is the expression difference is from large to small. The results are shown in Figure 7.

## 4. Conclusion

By 2019, 463 million adults aged 20 to 79 years worldwide had DM, approximately 15% to 25% of patients with type I diabetes and 30% to 40% of patients with type II diabetes developed renal complications and may progress to end-stage renal disease.^[6]^ However, the pathogenesis of DN is not yet clear, and the treatment is not comprehensive enough.

### 4.1. Analysis of core acting target results

In the PPI network analysis in this study, VEGFA, TNF, IL6, STAT3, TP53, ESR1, and JUN were tied for first place with a degree value of 22. VEGFA regulates vascular permeability and it is a key regulator of angiogenesis during the growth of solid tumors.^[7,8]^ It has been found that VEGF expression levels are significantly higher in renal epithelial cells and distal tubular epithelial cells in DN patients compared to the general population,^[9]^ which may be associated with increased renal vascular permeability leading to proteinuria. TNF is produced by monocytes and macrophages during acute immune responses and can promote cell proliferation, differentiation, cytokine production, apoptosis, and necrosis^[10]^; a close relationship between TNF and the development of DM has been demonstrated.^[11]^ IL6 is one of the most important inflammatory cytokines, while IL6 can bind to specific receptors to stimulate the Janus kinase/signal transducer and activator of transcription signaling pathway,^[12]^ plays a vital role in peripheral metabolic organs such as the adipose, pancreas, and immune system, and is a new potential therapeutic target for diabetes.^[13]^ STAT3 can affect cellular autophagy through different subcellular localization patterns. For example, cytoplasmic STAT3 inhibits cellular autophagy by sequestering eukaryotic initiation factor 2-*α* kinase 2 and other signaling molecules associated with autophagy.^[14–16]^ In addition, mitochondrial translocation of STAT3 inhibits oxidative stress-induced autophagy and may effectively protect the mitochondria from mitotic degradation.^[17–19]^ TP53 encodes proteins that respond to various cellular stresses and regulate the expression of target genes to induce cell cycle arrest, apoptosis, senescence, DNA repair, or metabolic changes^[20–22]^; for example, by downregulating the anti-apoptotic gene product Bcl-2, upregulates the pro-apoptotic gene product Bax, and other pathways to participate in the regulation of the apoptosis pathway.^[23]^ The ESR1 inhibits nuclear factor kappa B (NF-*κ*B) -mediated transcription of the IL6 promoter by reducing NF-*κ*B DNA-binding activity and displaces RELA/p65 and associated co-regulators from the promoter,^[24]^ thereby regulating inflammation, immunity, and stress responses. AP-1 (AP1), a transcription factor involved in JUN, is a recognized integrator of extracellular signals^[25,26]^ and plays a vital role in the treatment of various inflammatory lesions, transplant rejection, fibrosis, and organ damage.^[27]^ The remaining core target genes were mostly associated with inflammatory responses, cellular autophagy, immunity, oxidative stress, apoptosis, cellular senescence, and tumors.

### 4.2. Analysis of pathway and biological process

A total of 109 KEGG pathways were obtained in this study, and the enrichment results showed that TP treatment of DN mainly involved the following types of signaling pathways: DM-related, immune-related, and cell survival-related pathways.

DM-related pathways include the AGE-RAGE signaling pathway in diabetic complications and insulin resistance. The AGE-RAGE signaling pathway activates NF-*κ*B and causes the expression and release of large amounts of IL6 and TNF, which eventually causes chronic cell activation and tissue damage.^[28,29]^ In podocytes, the AGE-RAGE signaling pathway can stimulate VEGF, which increases vascular permeability and causes proteinuria.^[30]^ It also stimulates the production of TGF-*β*1, which leads to glomerular extracellular matrix production and tubular epithelial mesenchymalization^[31–33]^; induces enhanced MCP-1 expression and promotes glomerulosclerosis and tubulointerstitial fibrosis^[34]^; stimulates angiotensin II production or overexpression at renal component AT1 receptors to accelerate the progression of DM^[35]^; AGE-RAGE signaling pathway activates nicotinamide adenine dinucleotide phosphate oxidase, which in turn activates mitogen-activated protein kinase, extracellular regulated protein kinases, extracellular signal-regulated kinase [ERK1/2] ERK1/2 and P38 and other mediated signaling pathways via reactive oxygen species, resulting in the activation of NF-*κ*B by phosphorylation.^[36–38]^ TP may reduce renal damage and decrease urinary protein levels in DN patients through the AGE-RAGE signaling pathway and has a positive effect on blood glucose in DN patients through insulin resistance

Immune-related pathways include the Interleukin 17 (IL-17) signaling pathway, the Toll-like receptor signaling pathway, Th1 and Th2 cell differentiation, and the NF-*κ*B signaling pathway. The IL-17 signaling pathway can activate NF-*κ*B and induce NF-*κ*B-dependent cytokines to upregulate inflammatory gene expression^[39]^; IL-17 has pathogenic functions in immune-mediated glomerular diseases.^[40]^ IL-17 has also been found to limit the growth of fungi in the kidney, prevent kidney tissue damage, and protect kidney function during the transmission of candidiasis through the kallikrein (Klk) Kallikrein- Kinin system (KKS).^[41]^ The Toll-like receptor signaling pathway (TILs signaling pathway) is one of the earliest determinants of immune activation, which can activate TGF-*β*1, TNF, AT1, NF-*κ*B, etc, through cellular exogenous, intrinsic, and specific responses^[42,43]^ to induce a series of immune responses and thus have an impact on DN. Th1 and Th2 cell differentiation is influenced by various factors, such as antigen type and concentration, co-stimulatory molecules, cytokine concentration, immunoreactive hormones, transcription factors, and type of antigen-presenting cells. Disruption of the Th1/ Th2 balance can induce a range of autoimmune diseases and adversely affect pancreatic *β*-cells,^[44]^ thereby aggravating the condition of DN patients. The NF-*κ*B signaling pathway includes 3 main pathways: the canonical, alternative, and atypical pathways. After activation, it can combine with other pathways, such as the TILs signaling pathway, to cause inflammation and fibrosis in the kidneys.^[45,46]^ TP may treat DN by inhibiting local inflammation and excessive immunity in the kidney through the IL-17 signaling pathway, Toll-like receptor signaling pathway, Th1 and Th2 cell differentiation, NF-*κ*B signaling pathway, etc.

Cell survival-related pathways include the TNF, apoptosis, NF-*κ*B, PI3K-Akt, mTOR, and IL-17 signaling pathways. The TNF signaling pathway induces podocyte apoptosis by inducing overexpression of retinoic acid receptor responder 1 in podocytes.^[47]^ While mediating renal inflammation and fibrosis, the NF-*κ*B signaling pathway also aggravates renal injury and proteinuria by inducing apoptosis through oxidative stress.^[48]^ The PI3K/AKT signaling pathway is related to proliferation, differentiation, and apoptosis. It connects downstream mTOR signaling to the PI3K-AKT-mTOR pathway, which can induce autophagy and increase the renal oxidative stress response.^[49,50]^ The mTOR pathway can also exacerbate glomerulosclerosis by promoting DN mouse thylakoid proliferation in combination with other factors and pathways,^[51]^ which has been confirmed by in vitro experiments on human renal thylakoid cells.^[52]^ In addition, the IL-17 signaling pathway activates the mitogen-activated protein kinase pathway, which includes extracellular signal-regulated kinase, p38, and JUN N-terminal kinase, to induce apoptosis.^[39]^ TP may reduce renal injury and decrease proteinuria by reducing apoptosis and autophagy in renal cells by inhibiting the TNF, NF-*κ*B, PI3K-Akt, mTOR, and other signaling pathways.

### 4.3. Analysis of co-acting targets analysis

Analysis of the retrieved datasets found that TP inhibited the overexpressed CASP3, CASP8, CCNE1, CXCR4, GSK3B, HSPA5, Janus kinase 2, NFE2L2, PLAU, PTEN and PTGS2 in DKD, while upregulating ESR1 and TGFB1, which were repressed in DKD, promoting each gene restores its proper expression level, thereby treating DKD. The expression trends of some other genes under TP intervention were consistent with the trends in DKD, suggesting that TP is less likely to treat DKD through these genes. However, human is a complex whole, and the pathways and organismal responses associated with each gene are complex and variable. Therefore, a consistent trend does not mean that regulating the expression of these genes is ineffective, and further experimental verification is still needed. In addition, different disease stages, disease states, and individual responses to gene expression may also vary widely, and the effects of drugs on them will also be different. Perhaps this can be used to explain the poor efficacy of some patients in clinical practice.

### 4.4. Summary and prospect

At present, the construction of a drug-disease molecular network is rarely reported in DN. The application of molecular network science can help to further reveal the mechanism of TP for DN treatment and provide direction for further research on pharmacological, toxicological, and pharmacodynamic substance bases. In this study, the mechanism of TP in the treatment of DN was predicted based on network pharmacology and molecular docking, and it was found that the mechanism was mainly related to the reduction in the inflammatory response, immune suppression, inhibition of apoptosis, and inhibition of glomerulosclerosis. In addition, TP may have ameliorating effects on insulin resistance. However, this study only predicted the mechanism of TP for DN from the available internet data, lacking experimental and clinical validation, and failed to clarify the treatment protocol for the prescribed dose and duration of TP for DN. Therefore, further experimental validation and validation in a large sample, multicenter, randomized, controlled clinical trials are still needed.

## Author contributions

YG designed the study and wrote the paper. YL and ZG performed the research and analyzed the data.

**Conceptualization:** Ying Gao.

**Data curation:** Yingying liu.

**Formal analysis:** Ying Gao, Zhaoan Guo.

**Writing – original draft:** Ying Gao, Zhaoan Guo.
